# Pangenomes aid accurate detection of large insertions and deletions from targeted sequencing: the case of cardiomyopathies

**DOI:** 10.1186/s13073-026-01749-0

**Published:** 2026-08-26

**Authors:** Francesco Mazzarotto, Özem Kalay, Elif Arslan, Valeria Cinquina, Rachel J. Buchan, Valeria Bertini, Esmé Cavanagh, Deniz Turgut, Mona Allouba, Alaa Afify, Sarah Halawa, Pantazis Theotokis, Gungor Budak, Francesca Girolami, Alessia Azzu, Petra Peldova, Nik Matthews, Dudley J. Pennell, Jiri Bonaventura, Iacopo Olivotto, Elisabetta Pelo, Marina Colombi, Milan Macek, Paul J. R. Barton, Yasmine Aguib, Magdi Yacoub, Marco Ritelli, Massimo Gennarelli, H. Serhat Tetikol, Roddy Walsh, James S. Ware, Amit Jain

**Affiliations:** 1 Department of Molecular and Translational Medicine, Università degli Studi di Brescia, Brescia, Italy; 2 National Heart and Lung Institute, Imperial College London, London, UK; 3 Velsera Inc, Charlestown, MA USA; 4 Royal Brompton & Harefield Hospitals, Guy’s and St. Thomas’ NHS Foundation Trust, London, UK; 5 MRC Laboratory of Medical Sciences, Imperial College London, London, UK; 6 Aswan Heart Centre, Aswan, Egypt; 7 Meyer Children’s Hospital, IRCCS, Florence, Italy; 8 Cardiovascular Magnetic Resonance Unit, Royal Brompton Hospital, London, UK; 9 Department of Biology and Medical Genetics, Second Faculty of Medicine, Charles University and Motol University Hospital, Prague, Czech Republic; 10 Imperial BRC Genomics Facility, Imperial College London, London, UK; 11 Department of Cardiology, Second Faculty of Medicine, Charles University and Motol University Hospital, Prague, Czech Republic; 12 Department of Experimental and Clinical Medicine, University of Florence, Florence, Italy; 13 SOD Diagnostica Genetica, Azienda Ospedaliero Universitaria Careggi, Florence, Italy; 14 Genetics Unit, IRCCS Istituto Centro San Giovanni di Dio Fatebenefratelli, Brescia, Italy; 15 Bristol Myers Squibb, Cambridge, MA USA; 16 Cardiovascular and Genomics Research Institute, City St. George’s University of London, London, UK; 17 Department of Clinical and Experimental Cardiology, Amsterdam UMC, University of Amsterdam, Amsterdam, The Netherlands; 18 Hammersmith Hospital, Imperial College Healthcare NHS Trust, London, UK; 19 Meiosys Ltd, London, UK

**Keywords:** Pangenomes, Large variants, Targeted sequencing, Cardiomyopathy

## Abstract

**Background:**

Gene panels represent a widely used strategy for genetic testing in a vast range of Mendelian disorders. While this approach aids reliable bioinformatic detection of short coding variants, it often fails to detect many larger variants. Recent studies have recommended the adoption of pangenome references (as opposed to linear reference genomes like GRCh38) to augment detection of large variants from targeted sequencing, potentially providing diagnostic laboratories with the possibility to streamline diagnostic work-ups and reduce costs.

**Methods:**

Here, we analyze 1969 cardiomyopathy cases and 1805 controls sequenced with the Illumina Trusight Cardio panel using a pangenome-based workflow (GRAF) and five conventional orthogonal methodologies (GATK HaplotypeCaller, GATK-gCNV, ExomeDepth, Manta and Lumpy-SV) to detect variants ≥ 20 bp in size.

**Results:**

Following lab-based variant validation by means of PCR and Sanger sequencing, we show that GRAF conjugates higher precision and recall (F1 score 0.86) compared with other methods (F1 0-0.57) in detecting potentially pathogenic variants ≥ 20 bp from short-read panel data. Results were complemented by a comparison of the tools’ performance in detecting ground truth variants on reference sample HG002 from Genome In A Bottle, which confirmed GRAF to outperform other tools also on exome sequencing (F1 0.97 vs. 0-0.94). Notably, in the HG002 benchmark dataset, GRAF also showed slightly improved performance compared to GATK HaplotypeCaller in the identification of small variants (1–19 bp; F1 0.975 vs. 0.968).

**Conclusions:**

Our results indicate that pangenome-based workflows aid improved detection of large variants from targeted sequencing data in the clinical context and suggest that they may contribute to more unified variant detection frameworks for all-size genetic variants in the future.

**Supplementary Information:**

The online version contains supplementary material available at https://doi.org/10.1186/s13073-026-01749-0.

## Background

Large sequence variants are historically difficult to detect from short-read targeted sequencing assays like exome sequencing (WES) or gene panels, due to limitations intrinsic to these technologies [1, 2]. This is a relevant drawback in the clinical context, as – in spite of decreasing costs of genome sequencing (WGS) – short-read WES and targeted gene panels still represent the most widely used approaches for the genetic diagnosis of inherited conditions.

Routine adoption of (especially long-read) WGS would improve bioinformatic detection of large variants, including those in non-coding regions. However, targeted sequencing approaches such as gene panels are still characterized by markedly lower costs and – due to difficulties in interpreting non-coding variation in the clinical context – similar diagnostic yields [3–6].

Optical genome mapping represents an interesting option for cost-effective detection of large variants but is still not as widespread or approved for diagnostics and, in this context, clinical laboratories still rely on wet lab technologies such as MLPA and array CGH to characterize large genetic variation. While these approaches enable reliable detection of large duplications and deletions, they are characterized by low throughput and high costs, besides being laboratory intensive. Furthermore, these strategies don’t generally enable breakpoint resolution, not being informative on the exact locations where variants occur.

For these reasons, the availability of computational approaches enabling efficient large variant detection from short-read targeted sequencing assays would be highly advantageous for diagnostic laboratories, providing the possibility to reduce costs and streamline diagnostic work-ups. The numerous tools developed for this purpose are characterized by variable performance across datasets and variant classes, and identify variants exploiting one or more signals including read depth changes, the presence of split reads and discordant mapping of paired reads [7–9].

The different strengths and weaknesses characterizing every tool and signal type fostered the development of approaches combining multiple callers to increase accuracy [10–12]. However, the routine application of such pipelines entailing the usage of multiple tools often represent a hurdle in diagnostic laboratories without a dedicated bioinformatics team.

In this context, the advent of pangenome references (as opposed to single haplotype reference assemblies like GRCh38), potentially represented the beginning of a revolution as far as large variant detection from short-read targeted sequencing is concerned.

Pangenome references include the conventional reference genome as well as known genetic variation and alternative haplotypes, obtained from databases and cohorts [13]. This not only makes them variant-aware, with a consequent significant improvement of read mapping accuracy, but also renders them tailorable, for example to a given ancestry [14]. The augmented accuracy in read alignment translates into substantial improvements in variant identification, with this being especially pronounced for large variants [15]. As a result, the adoption of a pangenome reference can aid precise identification of large genetic variants across different organisms and sequencing technologies [16, 17].

However, the impact of the routine adoption of a pangenome-based approach in the clinical context has not been assessed to date. Here, we analyzed a multi-centric cohort of 1969 cardiomyopathy cases and 1805 controls sequenced with the Illumina Trusight Cardio panel [18] – widely used in the diagnostic context – with the Velsera pangenome-based GRAF pipeline [19] as well as five other approaches relying on orthogonal sources of evidence to detect variants ≥ 20 bp in size. While large variants are minor contributors to the cardiomyopathy disease burden [20–22], our performance comparison between GRAF and multiple other tools in detecting these variants from short-read targeted sequencing data provides the scientific community with findings applicable to any condition for which targeted sequencing panels are a genetic testing strategy.

In addition, we applied all 6 tools on WES reference sample HG002 from Genome In A Bottle (GIAB) to complement our cohort-based analyses with a well-characterized benchmark dataset.

Results on panel samples show GRAF to conjugate the highest recall with the complete absence of false positive calls (F1 = 0.86) and suggest how – with further refinements – pangenome-based bioinformatics approaches in the future may increasingly serve as effective screening tools, potentially reducing the reliance on wet-lab techniques in detecting large variants from gene panels. The analysis on HG002, for which the ground truth set of variants is known, confirmed GRAF to outperform the other tools on detecting variants ≥ 20 bp in size (F1 = 0.97 vs. 0-0.94) and to perform better than the widely adopted GATK Haplotype Caller in the detection of small variants as well (1–19 bp in size; F1 = 0.975 vs. 0.968).

These findings suggest how pangenome-based bioinformatics approaches could represent, in a not-so-distant future, a solution toward more unified frameworks to detect variants of all sizes, with a performance potentially rivaling that of wet-lab approaches. This would contribute to an important cost reduction for diagnostic laboratories, especially concerning conditions where large variants explain a consistent proportion of the disease burden.

## Methods

### Study cohorts

A total of 3774 samples sequenced on the Illumina Trusight Cardio panel, comprising 174 genes associated with inherited cardiac conditions, were obtained from Imperial College London (London, UK; *N* = 2150), the Aswan Heart Centre (Aswan, Egypt; *N* = 1273), the Careggi University Hospital (Florence, Italy; *N* = 219) and the Motol University Hospital (Prague, Czech Republic; *N* = 132). These comprised 1041 patients affected with hypertrophic cardiomyopathy, 928 affected with dilated cardiomyopathy and 1805 unaffected volunteers (UVOL). The number of samples each center contributed per cohort is available in Additional File 1: Table S1. Sequencing reads were aligned to the pangenome using the GRAF aligner for subsequent analysis with GRAF, and to the GRCh38 reference genome using BWA v0.7.17 for subsequent analysis with the other five variant callers, respectively. Details concerning enrolment and clinical characteristics of the analyzed sample sets are available in previous publications on the cohorts from London [23–25], Aswan [26, 27], Florence [28] and Prague [29].

### Analyzed genes

The analysis was focused on genes targeted by the Illumina Trusight Cardio panel and classified as moderate-, strong- or definitive-evidence genes for DCM (*N* = 18) and HCM (*N* = 14, 9 of which shared with DCM) by the gene-disease validity curations performed by the ClinGen consortium [30, 31]. Disease-specific gene sets were analyzed on DCM and HCM samples, while the full set of 23 genes was analyzed in UVOLs. *ACTC1*,* ACTN2*,* JPH2*,* MYH7*,* PLN*,* TPM1*,* TNNC1*,* TNNI3* and *TNNT2* were analyzed in both DCM and HCM cohorts, while *BAG3*,* DES*,* DSP*,* LMNA*,* NEXN*,* RBM20*,* SCN5A*,* TTN* and *VCL* were analyzed only in DCM and *CSRP3*,* MYL2*,* MYL3*,* MYBPC3* and *TRIM63* were analyzed only in HCM.

### Variant identification and data processing

All samples were analyzed with six methods to detect ≥ 20 bp variants, each selected for its specific strengths in the analysis of technically homogeneous short-read targeted sequencing data and leveraging complementary sources of evidence to infer presence of such variants:

- The pangenome-based Velsera GRAF pipeline v1.2 [19] – for this pipeline two disease-specific pangenomes (for DCM and HCM) were created upstream, with the aim of maximizing variant detection accuracy. Details concerning pangenome creation are listed in paragraph 2.6.
- GATK HaplotypeCaller v4.1 (GATK-HC) [32]; based on local reassembly - while not being a large variant caller per se, GATK-HC is a well-established method for detecting small variants and indels up to 50 bp in size, particularly at read depths > 50 ×  [33], and is widely used in both the research and the diagnostic settings. Data processing and variant calling were performed following the Broad Institute’s Best Practices for germline short variant identification.
- GATK-gCNV v4.6.2.0 [34]; advanced read depth-based caller implementing a Bayesian probabilistic model to infer copy number states across target intervals, jointly analyzing multiple samples to model read depth variability across datasets. GATK-gCNV is reported as a top-performing caller to detect copy number variants from gene panel data [34, 35]. Variant calling was performed following GATK Best Practices for germline copy number variant (CNV) discovery.
- Manta v1.6.0 [36]; large-variant caller combining paired-end and split-read evidence followed by local re-assembly, developed by Illumina and specifically designed for detecting large variants from Illumina short-read paired-end data.
- ExomeDepth v1.1.16 (ED) [37]; read depth-based copy number variant caller, well-suited for analyzing samples with highly correlated read depth profiles, like the ones we studied here. Since ED requires a set of reference samples for comparison, UVOLs were used as reference set when analyzing DCM and HCM patients. In the analysis of UVOLs, the cohort was split in half, performing the variant calling on 902 individuals and utilizing the remaining 903 as reference samples. The 902 UVOLs that underwent variant calling comprised those in which ≥ 1 variant of interest was detected by any other caller, for comparative purposes.
- LUMPY v0.2.13 [38]; large-variant caller integrating multiple types of signals into a probabilistic framework inferring variant presence, frequently included among SV callers in benchmarking studies. By jointly modeling multiple sources of information (including read-pair, split-read, and read depth), LUMPY inherently balances strengths and weaknesses of various data types.

Given the substantially higher number of variants detected in our cohorts by ED and GATK-gCNV in comparison with the other callers, we used ED’s Bayes Factor (BF) and GATK-gCNV’s Phred-scaled QUAL score to filter variants to a manageable shortlist of candidates for lab-based validation (see paragraph 2.7 for details).

### Benchmarking on GIAB reference sample HG002

To complement the cohort-based analyses with a benchmark dataset featuring a well-characterized truth set of variants and enable a more comprehensive assessment of the callers’ performance, including sensitivity, we analyzed reference sample HG002 from Genome In A Bottle (GIAB). Raw short-read paired-end Illumina WES data of HG002 were downloaded from the NCBI Sequence Read Archive (accession SRR2962669) and aligned to the GRCh38 reference genome and to the pangenome reference with BWA and the GRAF aligner, respectively. BAM files were processed with the 6 callers applied in this study, and each caller’s performance was assessed comparing variant calls passing quality control filters against truth set variants.

GIAB sample HG005 (accession SRR2962693, unrelated to HG002) and 168 individuals with cardiac sarcoidosis, sequenced with Illumina paired-end short-read WES, served as reference control dataset for ED and GATK-gCNV (both of which require reference samples for normalization and model construction). Benchmarking for GATK-HC, GRAF, LUMPY, and Manta was restricted to the intersection of the HG002 high-confidence regions and the target coordinates of the applied capture kit (Agilent SureSelect Human All Exon V5). For ED and GATK-gCNV, this target area was further intersected with the target coordinates of Agilent SureSelect Human All Exon V8, used for the sarcoidosis cohort, to ensure technical consistency.

Comparisons were performed using Truvari [39] for GRAF, GATKhc, Manta and Lumpy-SV, while Bedtools was used for ExomeDepth and GATK-gCNV due to differences in output formats. In addition, GRAF’s and GATKhc’s performance over the identification of small variants (1–19 bp in size) was assessed using RTG Tools (v3.13) vcfeval [40]. Commands used in the benchmarking analysis are provided in the Additional File 2: Supplementary Notes.

### Cohort variant filtering and comparison criteria

Variants ≥ 20 bp identified in DCM, HCM, and UVOL cohorts meeting the following inclusion criteria were retained for further analysis:

- Overlapping ≥ 1 exon or essential splice site in disease-specific genes for HCM and DCM (see paragraph 2.2) or in any of the analyzed genes for UVOL.
- PASS filter, combined with specific quality thresholds for GATK-gCNV and ED (see paragraph 2.7 for details).
- If in *TTN*, variants altering exons constitutively expressed in the heart (spliced into > 90% of the cardiac transcripts, Percent Spliced In > 90%) [23].
- Called in a sample with DNA available for lab-based validation.
- Variant size <1 Mb, as larger variants are often associated with syndromic phenotypes and fall outside the scope of isolated cardiomyopathy.

In line with established criteria [41], variant calls of the same type (gain vs. loss) within 200 bp of each other were merged up front to mitigate caller-specific over-segmentation and, in downstream analysis and lab validation, variants/variant calls were considered concordant if they showed ≥ 50% reciprocal overlap.

### Creation of disease-specific pangenomes

The GRCh38 linear reference was used as backbone for the pangenome’s construction, with high-confidence variants from the 1000 Genomes Phase 3 study [42], the Simons Genome Diversity Project [43] and the indels database by Mills et al. [44] added to the initial pangenome as alternative alignment paths. As previously described, cohort-specific variants (e.g. African-specific [14]) can also be used to augment the pangenome reference and further optimize it for cohort-specific analysis. For this reason:

- Sequencing reads from all 3774 samples were aligned to the pangenome using GRAF aligner v1.2.
- A preliminary set of large variants, detected by Manta v1.6.0 on the resulting BAM files, were added to the pangenome (variant details in Additional File 1: Table S2 and S3). This was done separately over two distinct sets of variants, enabling the creation of two disease-specific pangenomes:
  - The DCM-specific pangenome, augmented with variants detected in DCM and/or UVOL samples.
  - The HCM-specific pangenome augmented with variants detected in HCM and/or UVOL samples.

**Fig. 1:**
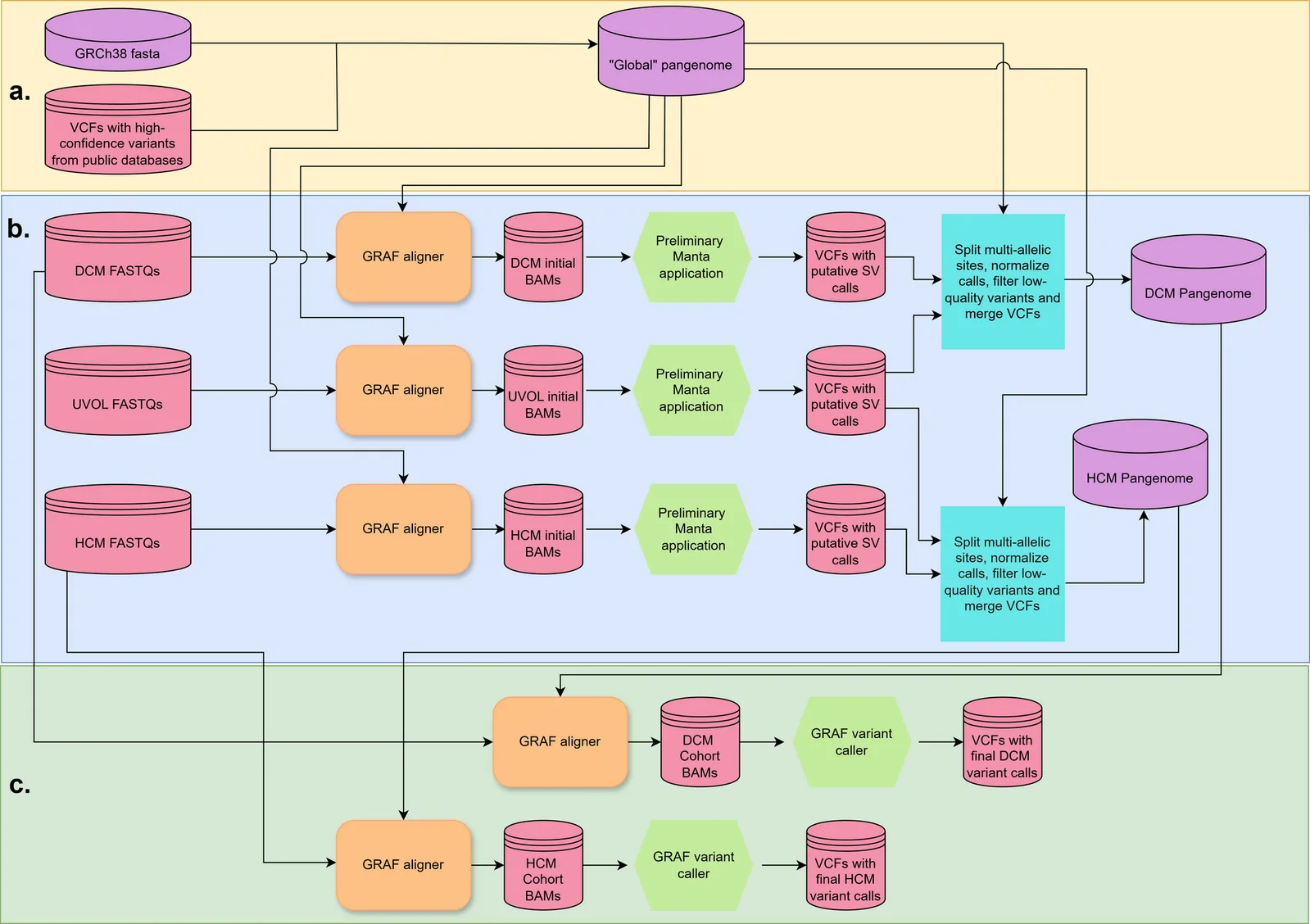
Workflow for pangenome construction and pangenome-based variant calling of sequencing samples. Panel a (yellow): **a** “global” pangenome reference is constituted by the GRCh38 linear reference (FASTA, used as a backbone) and high confidence variant calls from population cohorts (VCFs). Panel **b** (blue): raw data from DCM, HCM and UVOL cohorts are mapped against the pangenome to obtain pangenome-aligned BAM files. Preliminary, unvalidated variants detected by Manta on these BAM files are used to augment the global pangenome reference and construct disease-specific pangenomes. Panel **c** (green): raw data from DCM and HCM cohorts are re-aligned to the respective disease-specific pangenome. Subsequently, variant calling is performed with GRAF, which exploits variants posited by Manta (or any other large variant caller) to identify small and large variants. This was performed also on UVOL samples using both disease-specific pangenomes (not shown here). Purple cylinders represent reference (pan)genome files, pink cylinders represent other file types (stacked if multiple). Orange squares represent GRAF aligner applications. Green hexagons represent variant calling steps. Turquoise squares represent other operations (e.g. variant filtering)

Analyzing the 3774 panel samples with GRAF, DCM and HCM cohorts were processed using the DCM- and the HCM- specific pangenomes, respectively, while UVOLs were analyzed using both. A complete overview of the pangenome construction workflow and of the pangenome-based variant calling is provided in Fig. 1. Commands executed in running the GRAF pipeline are provided in Additional File 2: Supplementary Notes.

### Pre-processing of ExomeDepth and GATK-gCNV variant calls

ED generally favors sensitivity over specificity and consequently tends to call a high number of variants. Each variant is scored with a Bayes Factor (BF) reflective of ED’s confidence in the variant call, corresponding to the log10 likelihood ratio of the presence of the variant over the null hypothesis (its absence). Aiming at a manageable number of candidate variants to bring forward for lab-based validation while minimizing exclusion of true positives, we derived an empirical BF cutoff based on the burden of large variants (in the 32 analyzed genes) in the structural variants dataset of the Genome Aggregation Database (gnomAD SV v2.1, comprising 10,738 WGS samples and reporting variants > 50 bp in size). Initial calls made by ED comprised 71 distinct variants > 50 bp in 102 UVOLs (102 carriers in 902 analyzed UVOLs; 11.3%). We computed the burden of > 50 bp variants in the 32 analyzed genes in gnomAD SV (24 of 10,738 carriers; 0.22%) ranked ED’s calls by BF and, aiming at being conservative, applied a BF threshold yielding a burden of 0.5% of these variants in UVOLs (5 carriers in 902 UVOLs used as ‘cases’ in the ED analysis; BF ≥ 51.1).

A similar approach was applied to GATK-gCNV, which also produced an elevated number of initial calls (50 carriers in 1805 analyzed UVOLs; 2.7%). Applying the same criteria used for ED (filtering calls down to a conservative burden of 0.5%, i.e. 9 carriers in 1805 samples), we retained calls with QUAL ≥ 1463.19.

### Lab-based variant validation

Variants detected by one or more of the six tools and that passed the described filtering steps were brought forward for lab-based validation. Validation was performed by means of one or both of the following:

- PCR, used as a first-line assay to investigate the presence of variants.
- Sanger sequencing, which was directly used to sequence the variant through its entire length when smaller than ~ 500 bp in size or to sequence the breakpoint regions of longer variants, whenever PCR provided evidence in favor of their presence.

For duplications, PCR primers were designed also considering the possibility of a duplication coupled with an inversion event. In the analysis of validation results, a variant call was considered correct (i.e. a true positive) if the breakpoints – and the inserted sequence, in case of insertions – were correctly identified with no imprecisions by the variant caller.

Genomic regions of interest were PCR amplified by using optimized genomic primers. These primers were confirmed to lack known variants using the gnomAD database. PCR products were purified with ExoSAP-IT followed by bidirectional sequencing with the BigDye Terminator v1.1 Cycle Sequencing kit on the SeqStudio™ Genetic Analyzer System. Sequence analysis was performed with the Sequencher 5.0 software, and variants were annotated according to the Human Genome Variation Society nomenclature using the Alamut Visual Plus version v1.11 (SOPHiA GENETICS).

## Results

### Prioritized variants

Filtering initially prioritized 76 raw variant calls meeting the study’s inclusion criteria (section “Cohort variant filtering and comparison criteria”). The merging of neighboring raw calls (≤ 200 bp of each other), refined this set to 65 calls (Table 1), pertaining to 54 distinct mutational events across the three cohorts (full details in Additional File 1: Table S4). Following the filtering steps, LUMPY had the highest number of variant calls brought forward for validation (*N* = 22, 33.8% of the total), followed by Manta (16, 24.6%), ED (13, 20.0%), GATK-gCNV (9, 13.8%), GRAF (3, 4.6%) and GATK-HC (2, 3.1%). Raw variant calls excluded from further analysis due to lack of DNA availability, variant call size ≥1 Mb and ED- and GATK-gCNV-specific custom variant quality filters are listed in Additional File 1: Table S5. As far as GRAF variant calls are concerned, no difference in terms of prioritized variants was observed in the UVOL cohort when aligning against the HCM or the DCM-specific pangenome.

**Fig. 2:**
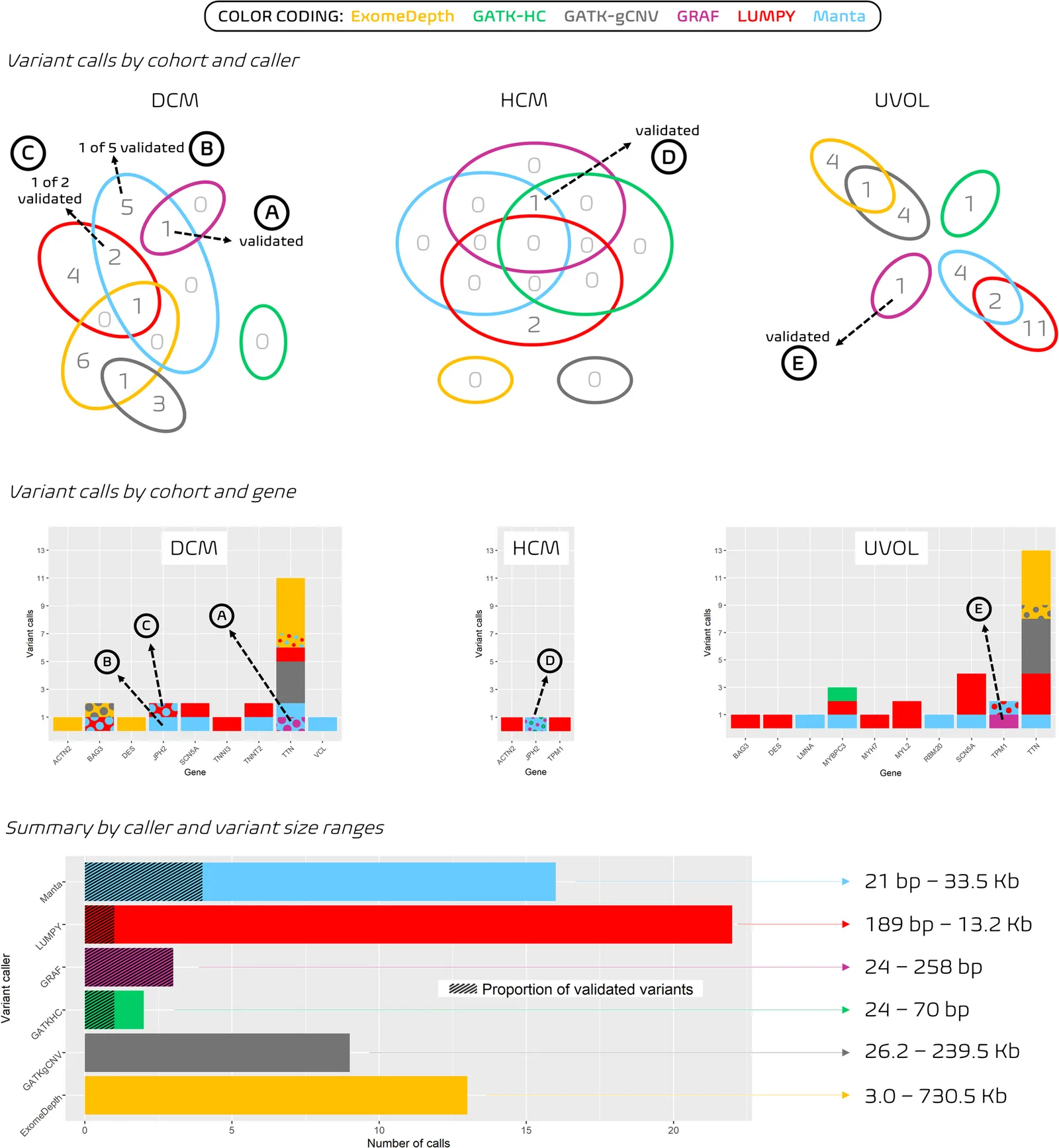
Overview of the 65 variant calls made by the six callers. The color coding outlined in the legend is consistent throughout the figure. Top: distribution of variant calls by cohort and caller, alongside details on which mutational events were validated by Sanger and/or PCR, labeled Ⓐ-Ⓔ (see text and Table 2). Middle: Distribution of variant calls by cohort and gene, with two- or three-color patterns indicating concordant calls by multiple callers. Bottom: total number of variant calls by each caller, alongside the proportion of variants confirmed by lab-based validation and variant calls’ size range

Distribution of variant call size varied significantly by tool (Table 1). Using each tool’s default settings, ED and GATK-gCNV uniquely called large variants (3-730 kb), with the latter also calling several variants >1 Mb, excluded up front. GATK-HC and GRAF called indels below 1 kb (24–258 bp), while Manta and LUMPY spanned both size ranges (21 bp-22 kb; Fig. 2). Sequence gain events (duplications/insertions) represented 53.8% (*N* = 35) of the total, though with stark differences between tools, with GATK-gCNV identifying only deletions (*N* = 9), and LUMPY calling almost exclusively duplications (19 of 22 calls; 86.4%).

### Validated variants and burden of potentially pathogenic ≥ 20 bp variants in DCM, HCM and in controls

Lab-based validation via Sanger sequencing and/or PCR (section “Lab-based variant validation”) confirmed five of the 54 predicted mutational events (9.3%), which were supported by the set of 65 prioritized variant calls listed in Table 1 (primers in Additional File 1: Table S6). In all five instances, the ground-truth variant - including exact breakpoints and sequence identity - was correctly identified by at least one tool. The callers exhibited markedly variable precision (defined as the proportion of true-positive calls over total prioritized calls), with GRAF achieving the highest (100%; 3 of 3 calls confirmed), followed by GATK-HC (50.0%; 1 of 2), Manta (25.0%; 4 of 16), and LUMPY (4.5%; 1 of 22). No prioritized calls were validated for GATK-gCNV (0 of 9) or ED (0 of 13). Full details of the validated variants are provided in Table 2, and wet-lab validation images (Sanger sequencing chromatograms and PCR gel images) are provided as Supplementary Figures. Based on these findings, we identified a burden of potentially pathogenic variants (≥ 20 bp) of 0.22% in DCM (2 variants in 928 samples) and 0.1% in HCM (1 in 1041), compared to a background rate of 0.05% in unaffected controls (1 in 1805). Notably, one DCM sample carried two validated variants in *cis* that, together, formed a single complex event.

**Table 1:** Variant calls (*N* = 65) prioritized for lab-based validation following the filtering steps and the intra-caller variant merging procedure detailed in section “Cohort variant filtering and comparison criteria”

Caller	*N*. of sample-variants	Cohort	Merged variant call ID (Supp. Table 5)	Gene	Type	Variant size (bp) and, [*n*. of merged calls]	Concordant variant calls by other tools
ED	13	DCM	4	*ACTN2*	gain	730,530	
			13	*TTN*	gain	76,636	
			15		gain	70,459	
			19		loss	46,319	
			24		gain	23,583	
			26		loss	3063	27, 28
			34	*DES*	gain	7608	
			45	*BAG3*	loss	25,687	44
		UVOL	7	*TTN*	loss	122,964	6
			12		gain	96,415	
			14		loss	71,562	
			20		loss	59,957	
			23		loss	26,971	
GATK-gCNV	9	DCM	9	*TTN*	loss	140,004	
			10		loss	137,775	
			11		loss	118,724	
			44	*BAG3*	loss	26,187	45
		UVOL	6	*TTN*	loss	239,526	7
			8		loss	155,681	
			22		loss	138,082	
			25		loss	135,065	
			29		loss	106,011	
GATK-HC	2	HCM	64	*JPH2*	loss	24	63, 65
		UVOL	50	*MYBPC3*	loss	70	
GRAF	3	DCM	17	*TTN*	gain	67	16
		HCM	63	*JPH2*	loss	24	64, 65
		UVOL	57	*TPM1*	loss	258	
LUMPY	22	DCM	3	*TNNT2*	gain	265	
			21	*TTN*	gain	275	
			28		loss	3427	26, 27
			41	*SCN5A*	gain	396	
			48	*BAG3*	loss	13,215	47
			59	*TNNI3*	gain	258	
			62	*JPH2*	gain	5969	61
		HCM	5	*ACTN2*	gain	457	
			58	*TPM1*	gain	329	
		UVOL	18	*TTN*	gain	399	
			30		gain	347	
			33		gain	287	
			35	*DES*	gain	276	
			36	*SCN5A*	gain	292	
			39		gain	220	
			40		gain	341	
			46	*BAG3*	gain	289	
			51	*MYBPC3*	gain	189	
			52	*MYL2*	gain	443	
			53		gain	340	
			54	*MYH7*	gain	378	
			56	*TPM1*	loss	22,384, [7]	55, see note ∆
Manta	16	DCM	2	*TNNT2*	gain	493	
			16	*TTN*	gain	134, [2]	17
			27	*TTN*	loss	3427	26, 28
			32	*TTN*	gain	22	
			37	*SCN5A*	loss	33,510	
			42	*VCL*	gain	901	
			47	*BAG3*	loss	13,218	48
			60	*JPH2*	loss	10,886	
			61	*JPH2*	gain	5967	62
		HCM	65	*JPH2*	loss	24	63, 64
		UVOL	1	*LMNA*	loss	21	
			31	*TTN*	gain	343	
			38	*SCN5A*	gain	399	
			43	*RBM20*	gain	570	
			49	*MYBPC3*	gain	254	
			55	*TPM1*	loss	22,401, [5]	56, see note ∆

Variant calls are considered concordant (rightmost column) only if of the same type and with a ≥ 50% reciprocal overlap (see section “Cohort variant filtering and comparison criteria”). Further details of each variant call are provided in Additional File 1: Table S4

∆ Within the merged call clusters for Manta (#55) and LUMPY (#56), individual raw calls were found to be concordant with the variant identified by GRAF (#57)

**Table 2:** Details concerning the 5 mutational events validated in the lab

Label in Fig. 2	Merged var. call ID(s)	Gene	Variant type	Variant length (bp)	Cohort	Centre	Variant caller(s)	Valid. strategy	Predicted effect	Notes
Ⓐ	16,17	*TTN*	gain	67	DCM	L	GRAF, Manta	S	Frameshift variant	♣
Ⓑ	60	*JPH2*	loss	10,886	DCM	L	Manta	P + S	Ⓑ and Ⓒ are carried in *cis* by the same sample, resulting in the dup. of exon 3 and part of exon 6, fused together	♥
Ⓒ	61,62	*JPH2*	gain	5968	DCM	L	Manta, LUMPY	P + S		
Ⓓ	63–65	*JPH2*	loss	24	HCM	F	GRAF, GATK-HC, Manta	S	Inframe deletion	
Ⓔ	57	*TPM1*	loss	258	UVOL	A	GRAF	P + S	Intronic deletion comprising the splice donor site	♠

Column “Centre”: L=London, A=Aswan, F=Florence. Column “Valid. strategy”: P = PCR, S=Sanger

♣ GRAF identified the exact variant. Manta’s prediction comprised two merged calls at the same locus (see Table 1 and Additional File 1: Table S4), with one matching the truth-set breakpoints but featuring a 1-bp sequence mismatch

♥ Manta precisely characterized this complex event (4918 bp) with two separate calls (10886 bp deletion + 5968 bp duplication). LUMPY identified the duplication, with proximal and distal breakpoints shifted by 1 bp and 2 bp, respectively, relative to the ground truth

♠ GRAF identified the exact variant. Among the merged raw calls at this locus (LUMPY *N* = 7, Manta *N* = 5), Manta correctly identified the variant, while the corresponding LUMPY call exhibited a 1-bp shift at the proximal breakpoint compared to the validated coordinates

### Performance benchmarking on GIAB WES sample HG002

The VCF truth set file from GIAB sample HG002 contains 45 high-confidence variants ≥ 20 bp and 40,362 high-confidence variants 1–19 bp overlapping the genomic regions to which the benchmarking analysis was restricted (section “Cohort variant filtering and comparison criteria”). GRAF yielded the best performance in detecting ≥ 20 bp variants (F1 score: 0.967) followed by GATK-HC (F1: 0.944) and Manta (F1: 0.890). LUMPY (F1: 0.072), ED and GATK-gCNV (both with F1: 0) were characterized by much lower precision and recall. The comparison between GRAF’s and GATK-HC’s performance in detecting small variants (1–19 bp) also showed GRAF to perform marginally better overall (F1: 0.975 vs. 0.968). Details on each tools’ performance are provided in Table 3. The full lists of variants called by each tool are available in the GitHub repository associated with this study (see the Data and Code Availability section).

Read alignment distributed over 64 CPUs and with 64Gb of available memory took 24 min when performed with BWA against GRCh38 and 12 min when performed with the GRAF aligner against the pangenome reference. Variant calling, including the necessary BAM file pre-processing steps when needed, and normalized to total CPU time taking into account the number of CPUs used, took 41 min for Manta, 144 min for GRAF, 373 min for GATK-HC and 422 min for LUMPY (ran using the *lumpyexpress* wrapper). CPU time estimates for ED and GATK-gCNV are heavily dependent on reference cohort size and the possibility to parallelize over multiple samples (for both tools) and over multiple genomic regions (for GATK-gCNV only). In our case, ED took 467 min with no parallelization (HG002 and 168 other WES samples processed sequentially), while GATK-gCNV took 458 min processing single samples in parallel whenever possible and splitting the exome into 21 regions when running GermlineCNVCaller (the most computationally demanding step).

**Table 3:** Performance metrics concerning each caller in the detection of variants ≥ 20 bp and 1-19 bp from WES data of GIAB sample HG002

	Caller	TP count	FP count	FN count	Precision	Recall	F1 score
Size: ≥20 bp	ED	0	282	45	0	0	0
	GATK-gCNV	0	204	45	0	0	0
	GATK-HC	42	2	3	0.955	0.933	0.944
	GRAF	44	2	1	0.957	0.978	0.967
	LUMPY	2	8	43	0.200	0.044	0.073
	Manta	40	5	5	0.890	0.890	0.890
Size: 1–19 bp	GATK-HC	38,447	618	1915	0.9842	0.9526	0.9681
	GRAF	39,740	1408	622	0.9656	0.9846	0.9750

TP=True Positive calls, FP=False positive calls, FN=False negative calls

## Discussion

In this study, we processed a total of 3774 technically matched, short-read panel-sequenced cardiomyopathy patients and controls with a pangenome-based method and five linear reference-based approaches, exploiting orthogonal types of signals, to detect variants ≥ 20 bp in size.

The main aim of this work was to evaluate whether pangenome-based strategies can aid detection of large insertions and deletions from targeted sequencing data, as gene panels and WES are still widely used in the diagnostic context. In addition, we were also interested in estimating the burden of potentially pathogenic variants ≥ 20 bp in DCM and HCM, accounting for the background rate in unaffected controls.

Overall, the low validation rate characterizing the mutational events predicted by the six callers (5 of 54, 9.3%) highlights the intrinsic difficulty in identifying these variants from short-read sequencing data. However, the tools applied were characterized by considerable performance variability, with precision ranging from 100% (GRAF, 3 of 3 predicted variants confirmed) to 0% (ED and GATK-gCNV, no variants confirmed among those predicted) and none of the tools able to detect all validated variants.

The initial number of calls made by ED and GATK-gCNV was substantially reduced up front, considering that the application of the empirically derived cutoffs described in section “Pre-processing of ExomeDepth and GATK-gCNV variant calls” caused the exclusion of 94.2% (227 of 241) and 91.3% (272 of 298) variants called in relevant genes, respectively. A relatively high number of false positive variant calls was somewhat expected especially for ED, given that ED generally favors sensitivity over specificity. However, the failure of both ED and GATK-gCNV in detecting any of the validated mutational events in our cohorts (in contrast with previously published analyses [35, 45–47]), as well as the high-confidence benchmark variants in HG002, is likely due to multiple factors. We hypothesize the main reason to reside in the critical importance of an appropriate selection of reference control samples, given that both these tools infer CNV presence through comparison of the analyzed individual with a set of reference controls. GATK-gCNV builds a cohort-wide read depth model using all provided samples and, while all our data has been produced with the same technology (Illumina paired-end sequencing) and the same gene panel (Trusight Cardio), specifically providing samples from the same sequencing run may improve its performance. In this work, however, we chose to analyze the cohorts as a whole, despite inclusion of multiple sequencing runs. This approach was chosen to better simulate a diagnostic laboratory environment, where timely analysis often necessitates processing samples as they arrive, rather than waiting for sufficiently large run-specific batches. Regardless, ED should be robust to this, as the tool is designed to automatically select the most highly depth-correlated samples from a cohort to minimize noise and, in this case, correlations were above the minimum of 0.97 advised to obtain reliable results (mean correlations: 0.992 for HCM and 0.998 for both DCM and UVOL samples). The reference control cohort used for HG002 also enabled meeting this criterion, with the read depth correlation computed by ED being 0.992. In ED’s case, another significant factor behind its underperformance may be represented by variant size and complexity. The majority of validated variants reported for ED in existing literature are large-scale CNVs [45, 48, 49], often spanning several kilobases (kb) to megabases, and in our case the average size of CNVs predicted by ED was 105 kb (range: 3-730 kb). Such large events were largely absent from our disease cohorts, which were characterized by smaller (Ⓐ,Ⓓ and Ⓔ; see Fig. 2; Table 2) or more complex (the combination of Ⓑ and Ⓒ; see Fig. 2; Table 2) events.

Manta, characterized by a validation rate of 25.0% (4 of 16 calls; Fig. 2) was the only tool that correctly identified a complex, kb-range duplication/deletion *JPH2* event (variant calls #60 and #61 in Tables 1 and Table 2), remarkably with exact breakpoints. This complex variant is the result of a 5967 bp duplication spanning part of exon 6 (in the 3’ untranslated region (UTR)), exon 5, exon 4 and part of exon 3 of *JPH2* combined with a larger 10,886 bp deletion, spanning a larger portion of the 3’ UTR and, again, exons 5, 4 and part of exon 3. Lab-based validation confirmed these two variants in *cis*, resulting in a net loss of 4919 bp and the creation of a chimeric 1997 bp sequence formed by parts of UTR exon 6, intron 2 and exon 3 (details in Supplementary Fig. 3). In addition, Manta correctly predicted events Ⓐ (67 bp duplication in *TTN* identified also by GRAF) and Ⓓ (24 bp deletion in JPH2 identified also by GRAF and GATK-HC), failing to identify only event Ⓔ among those validated. However, the five neighboring deletions called by Manta in the carrier of event Ⓔ (258 bp deletion in *TPM1;* Tables 1 and 2), subsequently merged into a 22 kb variant, included the correct event. Considering these results, recall emerges as Manta’s main strength, remarkably including also variants at the lower end of the ≥ 20 bp spectrum, despite default settings typically targeting variants > 50 bp. The tool’s high sensitivity was confirmed by the HG002 benchmarking, with Manta correctly identifying 40 of the 45 high-confidence truth variants. Notably, Manta’s false positive (FP) rate, on the other hand, exhibited a marked disparity between its application on panel-sequenced cohorts (12 of 16 calls being FP) and HG002 WES (5 of 45 calls being FP). This may suggest that differences such as the lower target density and the generally higher “off-target” noise associated with gene panels can result in a decreased reliability of breakpoint-based signals used by Manta to infer variant presence.

Although with slightly shifted breakpoints (1–2 bp) relative to the validated event, LUMPY was the only other tool partially detecting the complex *JPH2* variant called by Manta (successfully predicting the duplication part). In addition, similar to Manta, LUMPY made seven neighboring raw deletion calls in the carrier of event Ⓔ (Tables 1 and 2), including one matching the real variant. However, the tool’s overall performance was suboptimal on both panel data (1 validated variant call of 22; 4.55%) and HG002 WES (2 high-confidence variants identified among 45; 4.44%). These results likely reflect LUMPY’s heavy reliance on discordant read pairs and split-read signals, albeit not combined with local reassembly like in the case of Manta. The type of signatures LUMPY integrates into its underlying probabilistic framework are likely easier to resolve in WGS data, and this may explain the higher recall the tool achieves on WGS, though with substantial differences between duplications and deletions [50, 51].

GRAF was characterized by the absence of FP variant calls across the 3774 cohort samples, with the complex *JPH2* event being the only validated variant missed. The deletion component, in particular, was supported by a remarkably low proportion of reads (3 of 23 paired reads and 12 of 141 split reads). The current GRAF implementation works by looking for the best unique mapping of each read, even when this requires clipping the read ends. This strategy is generally optimal for most variants (regardless of size) but may be penalized when such a marginal share of the total reads supports the presence of the variant. In contrast, tools primarily relying on split-read evidence like Manta may be favored, yet at the cost of increasing FP calls. The sensitivity of split-read methods like Manta in edge cases like this (i.e. variants supported by a very low number of reads at the boundaries of the target region) is one of the reasons why we advise populating the initial “global” pangenome with raw variant calls from tools of this type (see paragraph 2.6 and Fig. 1). In contrast, GRAF is the only caller that correctly identified event Ⓔ (the 258 bp *TPM1* deletion) without making multiple FP calls in the region. This is an intronic deletion including the last base of *TPM1* exon 5 and its splice donor site and, while being at the boundary of the target region, GRAF realignment revealed a strong support in favor of its presence (336 reads of 607). Details concerning the wet lab-validation of this variant are provided in Additional File 3: Figure S4. GRAF’s performance on identifying variants ≥ 20 bp from HG002 WES data was characterized by remarkable precision (0.957) and recall (0.978), confirming it as the top performer among the tools tested here (F1: 0.967, Table 3).

GATK-HC’s performance on HG002 in detecting variants ≥ 20 bp was also noticeable (F1: 0.944), but the tool was characterized by a validation rate of 50% on our panel-sequenced cohorts, with 1 of 2 validated variant calls. The size of the validated event (event Ⓓ, 24 bp) compared with that of other confirmed variants (67 bp-10 kb) confirms GATK-HC’s focus on variants shorter than read length and especially with sizes in the lower end of the ≥ 20 bp range [33]. To evaluate the potential of pangenome-based approaches as a unique solution for all-size variant detection, we systematically compared GATK-HC and GRAF over the detection of small variants (1–19 bp) from HG002 WES. While both tools’ performance was remarkable, GRAF performed marginally better than GATK-HC (GATK-HC F1: 0.968 and GRAF F1: 0.975), with GRAF characterized by a higher FP call rate and by a lower false negative (FN) call rate compared with GATK-HC (FP call rate: 3.4% vs. 1.6%, respectively; FN call rate: 1.5% vs. 4.5%, respectively).

In terms of potentially pathogenic ≥ 20 bp variants in DCM and HCM, these results confirm their burden to be very low (≤ 0.2% for both HCM and DCM in this analysis). Limiting the comparison to the same genes screened here, a recently published analysis comprising the largest cardiomyopathy cohorts surveyed for CNVs to date detected comparable burdens to those observed in our cohorts (11/3550 (0.3%) vs. 2/928 (0.2%) in DCM, Fisher test *p* = 1 and 13/6779 (0.2%) vs. 1/1041 (0.1%) in HCM, Fisher test *p* = 1) [22]. In addition, the validated *TPM1* deletion detected in 1/1805 unaffected individuals may be reflective of the background rate in the population and – even if negligible – may translate into an even lower proportion of cardiomyopathy cases explained by large variants.

While we did not perform a cost estimation per se, CPU run time measurements showed GRAF to be particularly efficient compared with other tools considering its ability to identify large and small variants simultaneously. This suggests how pangenome-based approaches may lead to decreased costs and faster turnaround times for diagnostic laboratories.

## Conclusions

In conclusion, this work took cardiomyopathies as a use case to investigate the impact of pangenome-based approaches in detecting large variants from short-read targeted sequencing, constituting the most widely used approach in the diagnostic context. While findings reflect how identifying large variants from short-read targeted sequencing data remains challenging, they suggest how pangenome-based approaches like GRAF possess the potential to represent reliable solutions as all-size variant callers in the future for research as well as diagnostic settings, where targeted sequencing remains widely used.

We present this work in the hope that wider adoption of pangenome-based methods will improve the rate of positive diagnoses in patients suffering from genetic disorders, without imposing additional costs on healthcare providers.

## Limitations

Limitations of this study include the small number of validated variants, which constrains the ability to draw definitive conclusions regarding comparative tool performance in panel data. Additionally, our panel cohorts lack pre-defined positive control variants; therefore, we cannot exclude the presence of undetected variants missed by all six tools. However, the detailed analysis performed on reference sample HG002, with known variants, contributes to mitigating both weaknesses. The tools’ false-negative rates observed on HG002, combined with the historically limited burden of large variants reported in recent large-scale cardiomyopathy studies, suggest that our findings are representative of the true mutational landscape. Finally, while a sheer number of large variant callers exists, we selected a limited number of representative approaches based on orthogonal signals. Including more would have generated a number of candidates for validation difficult to manage, given the high false-positive rates typical of these methods and the large number of samples analyzed here.

## Supplementary Information

Below is the link to the electronic supplementary material.

Supplementary Material 1

Supplementary Material 2

Supplementary Material 3

## Data Availability

Pangenomes as well as all software required to reproduce the results we obtained with GRAF (and GATK-HC) are available in the following Docker images, hosted on the public Velsera Docker registry: • images.sbgenomics.com/ozem_kalay/gwgs-u:1.2 • images.sbgenomics.com/ozem_kalay/htslib-1-14:0 • images.sbgenomics.com/ozem_kalay/resolve_sv:0 • images.sbgenomics.com/ozem_kalay/bcftools-1-15-1:2 • images.sbgenomics.com/ozem_kalay/pangenome:0.12 • images.sbgenomics.com/ozem_kalay/gwgs-u:1.0rc3 • images.sbgenomics.com/ozem_kalay/pangenome:0.12.1GRAF is freely available through Velsera’s academic cloud platforms, including Cancer Genomics Cloud and Cavatica, as well as through the NHLBI BioData Catalyst platform) [52–55]. Restrictions apply for commercial use, please contact Velsera for terms and other details.All the scripts used in this study are available through the GitHub repository https://github.com/fmazzarotto/pangenome_targeted_seq_study_FMazzarotto and are permanently archived on FigShare (https://doi.org/10.6084/m9.figshare.32253483) [56].
