# Supplementary material for: Pangenomes aid accurate detection of large insertions and deletions from targeted sequencing: the case of cardiomyopathies
Source: Genome Med. 2026 Aug 26;18:132. doi: 10.1186/s13073-026-01749-0 (PMC13563831; doi:10.1186/s13073-026-01749-0)
Supplement: Supplementary file 1 [file 13073_2026_1749_MOESM2_ESM.docx]

**SUPPLEMENTARY NOTES**

**HG002 Benchmarking and Performance Evaluation**

*Definition of High-Confidence Evaluation Regions*

To ensure a rigorous comparison, benchmarking against the HG002 truth set (v4.2.1) was restricted to the intersection of the Agilent Human All Exon v5 target regions (the capture kit used for sequencing the HG002 WES sample and the reference controls) and the HG002 high-confidence genomic regions. The evaluation BED file was generated as follows:

bedtools intersect -a HG002_GRCh38_1_22_v4.2.1_benchmark_noinconsistent.bed -b S04380110_Covered.bed | bedtools sort | bedtools merge > exome-intersect_v5_highconfHG002.bed

*Benchmark Comparison*

The performance of GATK-HC, Manta, LUMPY, and GRAF in detecting variants ≥20bp was evaluated using Truvari (v4.1.0)., utilizing a ≥15bp buffer (--sizefilt 15) to capture borderline calls (e.g., a 19bp call representing a 20bp true deletion) to ensure that tool sensitivity was not artificially penalized by minor breakpoint shifts. The standard execution command was:

truvari bench --passonly --sizemin 20 --sizefilt 15 --sizemax -1 --bnddist -1 --dup-to-ins --includebed exome-intersect_v5_highconfHG002.bed -b HG002_GRCh38_1_22_v4.2.1_benchmark_norm.vcf.gz -c ${CALLER_OUTPUT}.vcf.gz -f Homo_sapiens_assembly38.fasta -o truvari_results_${CALLER}

For GATK-gCNV and ExomeDepth, Truvari could not natively parse the tool’s output, therefore comparison with the ground truth set was performed manually, intersecting variant intervals in the form of BED files. In addition, the cohort of 168 sarcoidosis WES samples provided to these tools as a technical reference control cohort was produced using a slightly different capture kit (Agilent Human All Exon v8).

For this reason, the exome-intersect_v5_highconfHG002.bed file (see above) was further intersected with the Agilent Human All Exon v8 target regions to produce overlap_AgiSSv5_HG002hc_AgiSSv8Sarcoid.bed.

For both tools, a two-step intersection strategy was used to determine sensitivity, first filtering for HG002 variants ≥20bp in the V5 ∩ V8 ∩ HG002-high-confidence regions:

bcftools view -T ${TARGDIR}/overlap_AgiSSv5_HG002hc_AgiSSv8Sarcoid.bed HG002_GRCh38_1_22_v4.2.1_benchmark_norm.vcf.gz | bcftools view -i 'strlen(ALT)>19 || strlen(REF)>19' -O z -o HG002_truthset_overlapBEDinExomeDepthWESsarcAnalysis_min20bp.vcf.gz

And subsequently intersecting these variants with those called by the tool in question, e.g. for ExomeDepth:

bedtools intersect -a HG002_truthset_overlapBEDinExomeDepthWESsarcAnalysis_min20bp.vcf.gz -b HG002_ED_calls_sarcWES.bed | wc -l

Of note, we ran ExomeDepth on HG002 three times, setting expected.CNV.length to 50000 bp (default), 500 bp and 50 bp when running command CallCNVs. This did not modify the benchmarking results, with ExomeDepth failing to detect HG002 ground truth variants.

The comparison between GRAF and GATK-HC over the detection of small variants (1-19bp) was performed with RTG Tools (v3.13) *vcfeval,* as follows (e.g. for GATK-HC), after having filtered HG002 truth set variants to keep those in the 1-19bp range with bcftools (bcftools view -i `strlen(REF) < 21 && strlen(ALT) < 21`):

rtg vcfeval --baseline=<HG0021to19bp> --bed-regions=exome-intersect_v5covered_highconfHG002.bed --calls=<gatkhc1to19bpcalls> --evaluation-regions=exome-intersect_v5covered_highconfHG002.bed --output vcfeval_results_max19bp_GATKhc --template=/rds/general/user/fmazzaro/home/WORK/Large_variants/resources/reference/Homo_sapiens_assembly38.sdf --no-roc --threads <threads>

**GRAF pangenome workflow commands**

GRAF was run on cohort samples as well as HG002 with the following commands:

*Read alignment*

aligner --cram_ref <cram_fasta> --vcf <graph_vcf> --reference <linear_reference> --markdup --fmt bam -q <cram_file> --read_group_platform <platform> --read_group_sample <sample_id> --sort --tmp ./sort.chunk.XXXXXX --sort_mem <mem> --hts_threads <htsthreads> --merge_threads <mthreads> --keep --index -o <output_name>

*Variant calling*

rasm -g <graph_vcf> -f <linear_reference> -b <input_bam> -v <output_vcf> -a <call_regions> -x all -o 1000000

*Results pre-processing*

bcftools norm -m -any <graf_raw_file> | bcftools view -i 'strlen(REF)>20 || strlen(ALT)>20' | bcftools filter --exclude 'AD_Ratio[1] < 0.20 || MBQ[1] < 15 || QD < 1 || MQRankSum < -12.5 || FS > 60' --mode x -o <graf_output> -Oz

This bcftools command is integrated into the GRAF default workflow as a standard filtering step.
