# Supplementary material for: Pangenomes aid accurate detection of large insertions and deletions from targeted sequencing: the case of cardiomyopathies
Source: Genome Med. 2026 Aug 26;18:132. doi: 10.1186/s13073-026-01749-0 (PMC13563831; doi:10.1186/s13073-026-01749-0)
Supplement: Supplementary file 2 [file 13073_2026_1749_MOESM3_ESM.docx]

**SUPPLEMENTARY FIGURES**

Figure S1 – details concerning the PCR- and Sanger sequencing-based validation of variant Ⓐ (67bp duplication in TTN). In the PCR image on the left: C=control, P=patient.

Figure S2 – details concerning the Sanger validation of variant Ⓓ (24bp inframe deletion in JPH2).

Figure S3 – details concerning the three PCRs conducted in the lab to validate the presence of the large, complex JPH2 event, resulting from the joint effect of variants Ⓑ (detected by Manta) and Ⓒ (detected by Manta and LUMPY) in a patient affected with dilated cardiomyopathy. These results confirmed one allele to be wild-type, and the other allele to carry the complex rearrangement. The net result on the mutant allele is the loss of a 4919bp sequence, but also the creation of a chimeric sequence formed by a proximal part of UTR exon 6 (763bp), intron 2 (85bp), exon 3 (89bp) and a distal part of exon 6 (1060bp).

Figure S4 – details concerning the lab-based validation of variant Ⓔ (258bp deletion in TPM1 detected in an unaffected volunteer).
